# Risk of postoperative nausea and vomiting in hip and knee arthroplasty: a prospective cohort study after spinal anaesthesia including intrathecal morphine

**DOI:** 10.1186/s12871-020-01154-z

**Published:** 2020-09-23

**Authors:** Antonio Moraitis, Magnus Hultin, Jakob Walldén

**Affiliations:** 1 Department of Surgical and Perioperative Sciences, Anaesthesiology and Intensive Care Medicine (Sundsvall), Umeå University, Sundsvall, Sweden; 2 Department of Surgical and Perioperative Sciences, Anaesthesiology and Intensive Care Medicine (Umeå), Umeå University, Umeå, Sweden

**Keywords:** Postoperative nausea and vomiting, Injections, Spinal, Prevention & Control, Arthroplasty, Morphine

## Abstract

**Background:**

The overall risk of postoperative nausea and vomiting after general anaesthesia is approximately 30% even with prophylactic medications. Studies exploring the risk after regional anaesthesia including intrathecal morphine are limited but indicate that intrathecal morphine is highly emetogenic and is additive to the PONV risk associated with other forms of anaesthesia. The aim of this observational study was to investigate the risk of PONV after spinal blockade combined with intrathecal morphine and to explore associations with patient and perioperative factors, including given PONV-prophylaxis. We hypothesized that a large number of patients in a clinical setting receive less prophylaxis than the recommendations in guidelines *(suboptimal prophylaxis)*, leading to a higher risk for PONV compared to those receiving adequate PONV prophylaxis.

**Methods:**

The study was conducted as a prospective observational cohort study regarding PONV in patients undergoing hip/knee replacement under spinal anaesthesia including intrathecal morphine. Patients were included at a county hospital in Sweden during April–November 2013 (*n* = 59) and September 2014–June 2015 (*n* = 40). One hundred eight patients entered the study with 99 patients analysed in the final cohort. Patients were followed the first three postoperative days with a questionnaire regarding PONV and peri- and postoperative data was collected. PONV risk is presented as the proportion of patients (%) with PONV and was related to the level of perioperative PONV-prophylaxis (suboptimal/optimal). Univariate analysis was used to analyse factors associated with PONV.

**Results:**

Forty-six patients (46%) experienced PONV during the 3-day study period whereof 36 patients (36%) until noon the first day after the procedure. 19/27 patients (70%) that received suboptimal PONV-prophylaxis experienced PONV compared to 27/72 (38%) that received optimal PONV-prophylaxis (*p* = 0.015). Further, female gender and/or a history of motion sickness were associated with an increased PONV-risk.

**Conclusions:**

There was a high risk for PONV after spinal anaesthesia including morphine. PONV risk was associated with the level of prophylaxis and with known risk factors for PONV. Our findings suggest that a more liberal use of PONV prophylaxis might be motivated.

## Background

The overall risk of postoperative nausea and vomiting (PONV) after general anaesthesia is reported to be approximately 30% even with prophylactic medications, but studies exploring the risk after regional anaesthesia including intrathecal morphine are limited [1, 2]. Hip and knee arthroplasty can be performed under regional anaesthesia using a spinal blockade, and postoperative pain can be reduced by adding intrathecal morphine [3, 4]. However, this method might be associated with a high risk of PONV [5, 6].

In Apfel’s model for assessing PONV risk, developed for general anaesthesia, significant risk factors are being female, non-smoker, having prior history of PONV and/or motion sickness, need of opioids for postoperative analgesia. If all factors are present, the risk of PONV can be as high as 80% [7].

Apfel’s risk score is often used for procedures under regional anaesthesia and prophylaxis given according to guidelines. Commonly used prophylactic drugs acting via different pharmacological targets are corticosteroids (anti-inflammatory), ondansetron (5-hydroxytriptamine 3 (5-HT_3_) receptor antagonist) and droperidol (dopamine D_2_ receptor antagonist), which are equally effective and each independently reduce PONV with approximately 25% [8]. Despite increased awareness and the introduction of new antiemetics, PONV is still a problem in the perioperative period. Risk stratification and a multimodal approach are key elements but are only effective if implemented and complied with, especially in high-risk patients [9–12]. Screening for PONV and a multimodal prophylaxis and treatment have a moderate evidence level and a strong recommendation grade [13].

The aim of this observational study was to investigate the risk of PONV in spinal blockade combined with intrathecal morphine and to explore factors associated with PONV with a focus on if the level of given PONV prophylaxis adhered to the guidelines. We hypothesized that a large proportion of patients receive less prophylaxis than recommended in a clinical setting, leading to a higher risk for PONV compared to those receiving adequate PONV prophylaxis.

## Methods

### Study design

The study cohort was part of a prospective observational cohort study regarding PONV. After obtaining written informed consent from the patients, data were collected from patient medical charts and through a standardized form with questions regarding PONV. The study was approved by the Regional Ethics Committee in Umeå, University Campus, Umeå, Sweden, on May 8, 2012 (Chairperson A. Iacobaeus, Dnr 2012/146-31 M).

### Study cohort

Our study cohort consisted of patients at Sundsvall Hospital, Sweden, undergoing hip/knee replacement under spinal anaesthesia including intrathecal morphine. Inclusion criteria were, besides the criteria for the cohort, age ≥ 18 years and being able to participate. Due to research resources, the inclusion was performed during two periods, April–November 2013 (*n* = 59) and September 2014–June 2015 (*n* = 40).

### Anaesthesia and PONV prophylaxis

The choice of anaesthesia, PONV prophylaxis and pain management was based on clinical routines and at the discretion of the attending anaesthesiologist. At the time of the study, the clinical routine for hip and knee arthroplasty was spinal anaesthesia with bupivacaine 15–20 mg, morphine 0.12 mg and clonidine 30 μg. PONV prophylaxis during anaesthesia were given according to local guidelines which stipulates that patients should be given a number of PONV prophylaxes equal to one less than the value of each patients Apfel-score. Drugs used for PONV prophylaxis were betamethasone 4 mg, ondansetron 4 mg, and droperidol 0.5–1 mg intravenously with a priority in that same order. Further, paracetamol (1 g × 4) and an oral depot opioid (oxycodone 10–20 mg/day) was given as base analgesics. Some patients also received a cox-2 inhibitor (etoricoxib 90–120 mg) in the premedication and some patients received gabapentin (600–900 mg/day) postoperatively for up to 7 days. As rescue analgesic, either intravenous morphine or peroral oxycodone were used. For all patients, an enhanced recovery pathway was used including early nutrition and mobilisation.

### Patient classification according to PONV risk and given prophylaxis

The simplified PONV risk score, developed by Apfel et al., was used to calculate the predicted the PONV risk [7]. Factors included in the model were female gender, non-smoking status, history of previous PONV and/or motion sickness, and use of postoperative opioids yielding a risk score of maximum 4. According to published guidelines, the optimal number of PONV prophylaxis to be given is related to the risk score [8]. To classify if patients had received PONV-prophylaxis according to our local guidelines, we (ourselves) defined *optimal prophylaxis* as the number of PONV prophylaxes needed being one less than the value of the risk score. When the number of prophylactic drugs given were less than *optimal prophylaxis*, patients were classified as having received *suboptimal prophylaxis*. Thus, *suboptimal prophylaxis* indicates that patients receives less PONV-prophylaxis than recommended. Our model is a simplification of the consensus guidelines published in 2014 [8], with the main difference that with four risk factors, where the guidelines states two or more interventions, we considered three interventions as optimal.

### Data collection

Preoperatively, patients were asked questions regarding risk factors for PONV. Postoperatively, patients answered a questionnaire with standardized questions regarding PONV at 2, 4, and 6 h after arrival at the recovery unit and at noon on postoperative days 1–3. The PONV questions were based on a validated scale for assessing PONV [14]. With the patient form, data regarding vomiting, retching, and nausea were obtained for each evaluation period, as well as the frequency of nausea [sometimes /often /most of the time /all the time] and if any rescue medications were given. Patients were also instructed to estimate any pain and to specify if any extra pain-relieving medicine had been taken. Further study data were obtained from the patients’ medical records and the perioperative charts.

### Primary outcome variables

PONV was defined as the presence of nausea and/or vomiting.

- Cumulative number of patients (%) with PONV over the observation intervals.
- Number of patients (%) with PONV during the postoperative intervals 0–2 h (hours), 2–4 h, 4–6 h, 6 h - Day 1, Day 1 - Day 2 and Day 2-Day 3.
- Number of patients with *suboptimal* or *optimal* PONV-prophylaxis.
- Number of patients (%) with PONV in relation level of prophylaxis (suboptimal/optimal).

### Secondary outcome variables

Patient and perioperative factors associated with PONV risk.

### Statistical methods

PONV risk is presented as the proportion of patients (%) with PONV, and a corresponding 95% confidence interval was calculated for PONV risk using the method recommended by Newcombe and Altman [15].

To explore factors associated with PONV risk, we performed univariate analysis on dichotomized data with Pearson’s chi-squared test and calculated odds ratios (ORs) with the corresponding 95% confidence intervals.

Data were entered in spreadsheets (Excel, Microsoft, Redmond, Washington, USA), and statistical analysis was performed with SPSS (IBM Corp. Released 2016, IBM SPSS statistics for Mac, Version 24, Armonk, New York, USA). Bar charts were created with MATLAB (Release 2018b, The MathWorks, Inc., Natick, Massachusetts, USA).

We did not perform any power calculation, although we considered the final study cohort of 99 patients to be an appropriate size for an observational study of a standardized procedure.

## Results

### Patient characteristics

Preoperatively, 109 patients were screened and asked for participation in the study, with 108 patients included. Seven patients did not receive spinal morphine and two patients had not answered any of the questions in the questionnaire, thus a final cohort of 99 patients were analysed.

The patients had a median age of 67 years (range 38–87 years), and 57% were female. Seventy-eight patients (79%) underwent hip arthroplasty and 21 (21%) underwent knee arthroplasty. The median dose of intrathecal morphine was 0.12 mg (range 0.08–0.20), and all patients received 30 μg intrathecal clonidine. Fifty-five patients (55%) had a high predicted risk for PONV (Apfel-score 3 or 4). For further patient characteristics see Table 1.

**Table 1:** Patient characteristics (*n* = 99)

	Value
Gender, *female sex*	42 (42%)
Age, *years*	67 (37–87)
BMI, *m*^*2*^*kg*^*−1*^	28 (19–43)
ASA-class	
1	26 (26%)
2	60 (61%)
3	13 (13%)
Smokers	1 (1%)
History of motion sickness	19 (19%)
Previous PONV	19 (19%)
Preoperative treatment with opioids	12 (12%)
COX-2 inhibitor in premedication	9 (9%)
Apfel-score	
0–1	0 (0%)
2	44 (44%)
3	35 (35%)
4	20 (20%)
Hip prosthesis surgery, *numbers*	78 (79%)
Knee prosthesis surgery, *numbers*	21 (21%)
Spinal anaesthesia combined with intrathecal morphine and clonidine	99 (100%)
Duration of anaesthesia, *minutes*	170 (105–336)
Duration of surgery, *minutes*	95 (57–256)
*PONV prophylaxis*	
Betamethasone	93 (94%)
Ondansetron	54 (55%)
Droperidol	20 (20%)
Number of prophylaxes given	
0	3 (3%)
1	45 (45%)
2	31 (31%)
3	20 (20%)
Rescue antiemetics until noon Day 1	27 (27%)
Gabapentin included in postoperative medication	36 (36%)

Values are presented as numeric values (% of total) or median (SD). *ASA* American Society of Anaesthesiologists Classification, *PONV* postoperative nausea and vomiting, *BMI* body mass index. PONV, number of prophylaxis given: Of those receiving only 1 prophylactic drug, 42 patients received betamethasone and 3 received ondansetron. For those given two prophylaxis, all received betamethasone and ondansetron. No missing data except BMI of one patient

### PONV prophylaxis

Ninety-four patients (94%) received betamethasone, 53 (54%) received ondansetron, and 20 (20%) received droperidol as PONV prophylaxes. Of those given only one prophylactic drug, 42 patients received betamethasone and 3 received ondansetron. The 31 patients given two prophylaxes all received betamethasone and ondansetron.

Twenty-seven patients (27%) received suboptimal prophylaxis in relation to their PONV risk score, and there was a significant difference between women and men in given suboptimal prophylaxis (45% [19 of 42 women] vs. 14% [8 of 57 men], *p* < 0.001; Table 2).

**Table 2:** Proportion of males vs. females receiving suboptimal prophylaxis

	***Men***	***Female***	*p-value*
	*(n = 57)*	*(n = 42)*	
**Suboptimal prophylaxis** *(n = 27)*	8 (14%)	19 (45%)	
**Optimal prophylaxis** *(n = 72)*	49 (86%)	23 (55%)	p < 0.001

*p*-value for comparisons between gender and suboptimal or optimal prophylaxis with Pearson’s chi-squared test

The risk of PONV in relation to the number of PONV-risk factors and the amount of PONV-prophylaxis given are presented in Table 3.

**Table 3:**
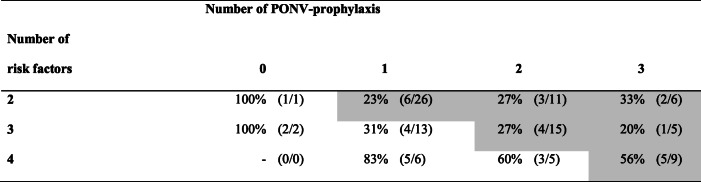
Risk of PONV (%) at 0–24 h in relation to PONV risk factors and number of PONV-prophylaxis given

Values are risk of PONV (number of patients with PONV in subgroup / total number of patients in subgroup). Grayshaded subgroups are considered given optimal prophylaxis in accordance to the definitions in the study

### Cumulative PONV risk

**Fig. 1:**
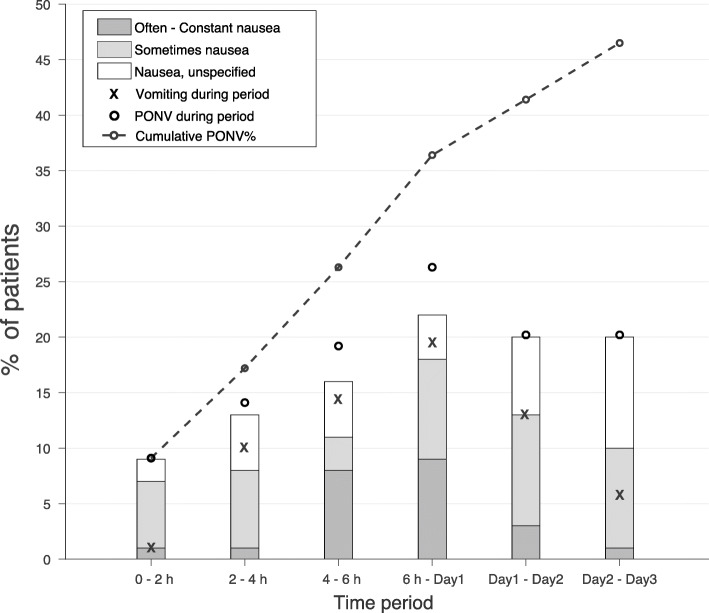
Cumulative risk of PONV and risk of nausea (including frequency), vomiting, and PONV during the different observation periods. Bars represent risk for nausea, and grey parts of the bars represent nausea that affected the patient’s activity (getting out of bed, moving in bed, walking normally, and eating or drinking) and was graded as sometimes (light grey) and often/constant (dark grey). The dashed line shows the cumulative incidence of PONV and accounts for the total number of patients with any event of nausea or vomiting

During the first 2 h, 9% (CI: 5–16%) experienced PONV with an increase to 26% (CI: 19–36%) in the first 6 h. At Day 1, 36% (CI: 28–46%) had experienced PONV, and at Day 3 46% (CI: 37–56%). Further details of PONV risk over time including each observation period are presented in Fig. 1 and Table 4.

**Table 4:** PONV risk in relation to the level of prophylaxis

Number of patients (%) with PONV	All patients (*n* = 99)	***Suboptimal PONV prophylaxis*** *(n = 27)*	***Optimal PONV prophylaxis*** *(n = 72)*	*p-value*
**0 h–6 h**	26 (26%)	9 (33%)	17 (24%)	*p* = 0.44
**6 h–Day 1**	26 (26%)	12 (44%)	14 (19%)	*p* < 0.05
**Day 1–Day 2**	20 (20%)	12 (44%)	8 (11%)	p < 0.01
**Day 2–Day 3**	20 (20%)	8 (30%)	12 (17%)	*p* = 0.17
**0 h–Day 1**	36 (36%)	15 (56%)	21 (29%)	p < 0.05
**0 h–Day 3**	46 (46%)	19 (70%)	27 (38%)	p < 0.01

*PONV* postoperative nausea and vomiting; comparison between suboptimal and optimal prophylaxis with Pearson’s chi-squared test. Data collected with a standardized questionnaire answered at 2, 4 and 6 h after arrival at the recovery unit and at noon on postoperative days 1–3. Risk of PONV during different postoperative periods after hip or knee arthroplasty under spinal anaesthesia including intrathecal morphine. Day 1 = first day after surgery

### Factors associated with PONV-risk

A higher incidence of PONV was observed during the first day among those who received suboptimal prophylaxis. On the third postoperative day, 70% of patients with suboptimal prophylaxis had experienced any PONV compared to 38% with optimal prophylaxis (*p* < 0.01; Table 4).

Being female (OR 2.33 (1.01–5.38)), having previous history of PONV (OR 5.37 (1.82–15.8)), and suboptimal PONV prophylaxis (OR 3.04 (1.22–7.57)) had higher odds for PONV until noon Day 1 (Table 5).

**Table 5:** Factors associated with risk for PONV until noon the first postoperative day

	Number of	Number of patients	Unadjusted OR	
	patients	with PONV (%)	(CI)	***p***-value
Gender				
Female	42	20 (48%)	2.33 (1.01–5.38)	0.046
Male	57	16 (28%)		
BMI				
< 35 kg/m^2^	88	30 (34%)	1.93 (0.52–7.20)	0.32
≥ 35 kg/m^2^	10	5 (50%)		
History of PONV				
Yes	19	13 (68%)	5.37 (1.82–15.8)	0.003
No	80	23 (29%)		
History of motion sickness				
Yes	19	8 (42%)	1.35 (0.49–3.75)	0.56
No	80	28 (35%)		
Non-smoker				
Yes	98	36 (36%)	NA	0.184
No	1	1 (100%)		
Preoperative treatment with opioids				
Yes	12	4 (33%)	0.90 (0.24–3.08)	0.81
No	87	32 (37%)		
COX-2 inhibitor in premedication				
Yes	9	3 (33%)	0.86 (0.21–3.68)	0.84
No	90	33 (37%)		
Suboptimal PONV prophylaxis				
Yes	27	15 (56%)	3.04 (1.22–7.57)	0.015
No	72	21 (29%)		
Type of prosthesis surgery				
Hip	78	27 (35%)	0.71 (0.26–1.88)	0.49
Knee	21	9 (43%)		
Intrathecal morphine, dose				
80–100 μg	11	5 (45%)	1.51 (0.42–5.42)	0.79
120 μg	76	27 (35%)	reference	
140–200 μg	12	4 (33%)	0.91 (0.25–3.29)	
Gabapentin given postoperatively				
Yes	36	13 (39%)	1.22 (0.52–2.88)	0.66
No	63	23 (35%)		
Given oral or parenteral opioids until noon Day 1				
Yes	97	35 (36%)	0.56 (0.03–9.3)	0.68
No	2	1 (50%)		
Maximal NRS ≥5 for pain until noon Day 1.				
Yes	39	17 (44%)	1.67 (0.72–3.84)	0.23
No	60	19 (32%)		

Number of patients is the total number in the subgroup. PONV risk is presented as the number of patients in the subgroup with PONV (%). Unadjusted OR, unadjusted odds ratio; *CI* 95% confidence interval, *NRS* Numeric Rating Scale, *Day 1* first day after surgery, *NA* not available for calculation

## Discussion

The main finding in our observational study in patients receiving intrathecal morphine was a high risk of PONV. The cumulative risk during the first three postoperative days showed that nearly half of the patients experienced PONV. Every fourth patient were given less PONV-prophylaxis than the recommendations in general guidelines, and these patients had an almost doubled risk for PONV. Further, females were given less prophylaxis to a greater extent and also had a higher risk of PONV.

The PONV risk found in our cohort are in accordance with previous studies. A meta-analysis evaluating intrathecal morphine (0.05–0.25 mg) in caesarean section found an overall PONV-risk of number needed to harm (NNH) of 6.3 for nausea and 10.1 for vomiting [5]. Another study investigating side effects in a randomized, double-blind, dose-response study concluded that nausea was present at even low doses of intrathecal morphine (0.015 mg) with an risk of 56% compared to a control group with a risk of 4% [6]. The absolute risks vary between studies and might be dependent on study settings and PONV prophylaxis, but many studies with intrathecal morphine report high risk for PONV [16–19].

Almost 80% of our patients *with PONV* during the three-day study period presented with their first episode of PONV during the first 24 h, indicating that there should be an awareness regarding symptoms of PONV and readiness for rescue treatments during the first postoperative day.

The third consensus guidelines from 2014 recommended that PONV-prophylaxis should be given according to individual PONV-risk and is based on the presence of PONV risk factors. With a higher risk score, the number of prophylactic interventions is increased [8]. The guideline states, that with 0, 1, 2, 3 or 4 risk factors, the minimum number of prophylactic agents to be 0, 0, 1, 2, 2, respectively. We simplified the model to a linear relationship and defined optimal prophylaxis as at least 0, 0, 1, 2, 3 agents with 0, 1, 2, 3, or 4 risk factors, respectively. The small differences between the models are that high-risk patients with four risk factors should be given three prophylaxis with our model, whereas the consensus guidelines recommend a minimum of two prophylaxis. As the highest risk for PONV were found in patients with four risk factors (Table 3), the results indicates that we used an acceptable model as there were differences between patients with three and four risk factors. Patients with four risk factors might be considered more prophylaxis than patients with three risk factors.

We found that almost one third of our patients were given *suboptimal* PONV-prophylaxis, even though there were local guidelines stating the optimal level of prophylaxis. Further, patients with suboptimal prophylaxis had an almost doubled risk for PONV. We cannot conclude if it was the suboptimal prophylaxis per se, the factors the classification was based on (i.e. female gender, previous PONV) or other unknown factors that actually caused the increased risk. Our findings agree with others that, even if guidelines are implemented in a clinical setting, there are major difficulties for clinicians to adhere to the guidelines [20]. A recent study showed that simplification of the risk assessment with a guidance to prophylaxis increased the number of patients receiving adequate PONV-prophylaxis and reduced the risk for PONV [21, 22].

We found that female gender and previous PONV were associated with a higher risk of PONV. Known risk factors for PONV are female gender, non-smoker, history of PONV or motion-sickness, and postoperative use of opioids [7]. With only one patient being a smoker and intrathecal opioids being given to all patients, it was not possible to evaluate the latter factors. Based on our findings, we suggest that factors associated with increased PONV risk after general anaesthesia might be relevant for predicting PONV risk after regional anaesthesia when intrathecal opioids are used as an adjuvant.

The use of opioids is one of the main factors that contribute to PONV [23]. All of our patients received intrathecal morphine, most of the patient’s postoperative oral opioids and some rescue opioids. With our observational study design, it was not possible to evaluate the effect of each type of opioid administration. However, patients with severe pain had a tendency to a higher PONV-risk and we might speculate that this is due to an increased number of opioids given to these patients.

As our study was an observational study, we did not interfere with the treatments given to the patients. Even if there were local guidelines, patients received different postoperative analgesic and we found differences in the doses of intrathecal opioids, some patients were given Cox-2 inhibitors before the procedure and one third of the patients received oral gabapentin postoperatively. We did not find any major association to an altered risk for PONV with these factors.

Our primary outcome variable (PONV) were based on reported events of nausea and vomiting. Except reporting the frequency of PONV, we did not put the PONV in the overall context of postoperative recovery. Today there are several validated tools to follow the postoperative quality of recovery, for example QoR-15 [24, 25], that includes several domains of recovery. Further, there are other factors, like fluid management and ambulation [26], that were not included in our study protocol and that may have impact on PONV. In future studies regarding PONV we believe it is of value to include these factors and an extensive quality of recovery tool.

A weakness in our study could be missing data, even though it was a prospective design. We might have missed PONV events that had not been identified or documented on the questionnaires, and thus the “true” result might be a higher risk for PONV. Further, there might be missed documentation of given PONV prophylaxis resulting in classification as suboptimal instead of optimal prophylaxis. This might have had an impact on the number of patients with suboptimal prophylaxis, but this would not have affected the observed high risk of PONV observed in our study.

The high risk of PONV in our study might suggest that patients undergoing hip and knee arthroplasty, including intrathecal morphine in the analgesic regime, might need more PONV prophylaxis. A main argument against a more liberal approach to PONV prophylaxis is an increased risk for side effects from the prophylactic drugs [2, 8]. However, antiemetics are generally well tolerated [27], and the benefits from adequate prophylaxis are desirable for patients, including increased well-being and a smoother postoperative recovery. Currently there is not enough evidence to make any firm conclusions, and more clinical studies are needed to evaluate the effects of liberal PONV prophylaxis.

Recently, the fourth consensus guidelines for the management of PONV were published [12]. The major change is a more liberal approach to PONV-prophylaxis with a recommendation to give two agents to patients with 1–2 risk factors and three or four agents when more than two risk factors are present. If generalised, the new guidelines propose that patients with any risk factors for PONV should be given 1–2 additional prophylactic intervention compared to our model used in this study. Our conclusion that more prophylaxis might be needed, are in line with these new recommendations.

## Conclusion

The risk for PONV after spinal morphine was high even with prophylaxis, and the increased risk was associated with known risk factors for PONV (female, previous PONV) and when a lower amount of PONV prophylaxis was given in relation to the risk. Our findings suggest that a more liberal use of PONV prophylaxis might be motivated.

## Data Availability

The datasets used and analysed during the current study are available from the corresponding author on reasonable request. The dataset will be edited to comply with necessary precautions to preserve individual privacy before being released.

## Abbreviations

ASA-class: American Society of Anesthesiologists classification
BMI: Body mass index
CI: Confidence interval
h: Hours
kg: Kilogram
mg: Milligram
μg: Microgram
NA: Not available
NNH: Number needed to harm
NRS: Numeric rating scale
OR: Odds ratio
PONV: Postoperative nausea and vomiting
QoR-15: Quality of recovery-15
SD: Standard deviation
